# Selective solvent filters for non-aqueous phase liquid separation from water

**DOI:** 10.1038/s41598-020-68920-4

**Published:** 2020-07-20

**Authors:** Tatianna Marshall, Klaudine M. Estepa, Maria Corradini, Alejandro G. Marangoni, Brent Sleep, Erica Pensini

**Affiliations:** 1 School of Engineering, University of Guelph, 50 Stone Road East, Guelph, ON N1G 2W1 Canada; 2 Food Science Department, University of Guelph, 50 Stone Road East, Guelph, ON N1G 2W1 Canada; 3 Food Science Department, Ontario Agricultural College, University of Guelph, 50 Stone Road East, Guelph, ON N1G 2W1 Canada; 4 Civil and Mineral Engineering Department, University of Toronto, 35 St George St, Toronto, ON M5S 1A4 Canada

**Keywords:** Environmental impact, Chemical engineering

## Abstract

Injectable filters permeable to water but impermeable to non-polar solvents were developed to contain non-aqueous phase liquids (NAPL) in contaminated aquifers, hence protecting downstream receptors during NAPL remediation. Filters were produced by injecting aqueous solutions of 0.01% chitosan, hydroxyethylcellulose and quaternized hydroxyethylcellulose into sand columns, followed by rinsing with water. Polymer sorption onto silica was verified using a quartz-crystal microbalance with dissipation monitoring. Fluorescence and gas chromatography mass spectroscopy showed low ppm range concentrations of non-polar solvents (e.g., hexane and toluene) in water eluted from the filters (in the absence of emulsifiers). The contact angles between polymer-coated surfaces and hexane or toluene were > 90°, indicating surface oleophobicity. Organic, polar solvents (e.g. tetrahydrofuran and tetrachloroethylene, TCE) were not separated from water. The contact angles between polymer-coated surfaces and TCE was also > 90°. However, the contact area with polymer coated surfaces was greater for TCE than non-polar solvents, suggesting higher affinity between TCE and the surfaces. Emulsifiers can be used to facilitate NAPL extraction from aquifers. Emulsion separation efficiency depended on the emulsifier used. Emulsions were not separated with classical surfactants (e.g. Tween 20 and oleic acid) or alkaline zein solutions. Partial emulsion separation was achieved with humic acids and zein particles.

## Introduction

Petroleum hydrocarbons (e.g. toluene) are common soil and groundwater contaminants because of their historical and current use in industry^1^. Oil spills in surface waters can also occur during transport by boat, threatening surface water bodies and ecosystems in their proximity^2–4^. In 1969, the oil well blowout in Santa Barbara, California released 4.1–4.6 million barrels of oil^5,6^. Since then, American waters have been contaminated by more than 44 oil spills (each over 420,000 US gallons)^7^. Spills have also occurred in Greece (where the Agia Zoni II released over 2,500 tonnes of oil in 2017^8^), in India (where the Ennore spill released 160 tonnes of oil^9^) and in east China (where the Sanchi oil tanker released 136,000 tonnes of natural-gas condensate in 2018^10^).

In addition to sorbents^11,12^, separation sponges have been developed to clean-up oil spills in surface waters. Separation sponges can be attached onto suction ports on boats, to selectively extract oil from surface waters^13^. Oil–water separation sponges are produced using different methods, including dip coating, chemical vapor deposition, in situ chemical reaction, wet chemical reaction, thermal treatment, polymerization, electroless deposition, or carbonization^13^. Different materials have been used to produce sponges for oil–water separation. For instance, graphene/polyurethane sponges have been obtained by in situ polymerization of *N*-methylpyrrolidone in the presence of graphene^14^. These sponges have been used to selectively extract hexane, crude oil and engine oil floating on water^14^. Polystyrene/Fe_3_O_4_/graphene aerogel composites with a reticulated graphene structure were used to sorb diesel oil and were subsequently retrieved from water using a magnet^15^. Polyacrylamide coated meshes were used to separate oil from water, by allowing exclusively water to flow through them^16^. Polyurethane foams instead allowed for the flow of crude oil, but not water^17^. Hydrophobic sponges have been obtained through the dip-coating method using SiO_2_ nanoparticle/polydimethylsiloxane coating and used to selectively extract hexane from water using a lab-scale pumping apparatus^18^. Superhydrophobic melamine sponges were obtained by coating with polydimethylsiloxane through thiol–ene click reaction and used to separate water from different oils, including soybean oil, pump and machine oil, diesel, cyclohexane and acetone^19^. Cotton fibers were modified to separate oil from water through the vapor phase deposition of polyaniline and fluorinated alkyl silane on cotton fabric^20^. Graphdiyne foams for oil–water separation were obtained using commercial copper foams as both the catalyst for the graphdiyne synthesis and 3D substrate to support graphdiyne nanostructures^21^. Graphene aerogels for oil–water separation were also developed^22^. In situ growth of transition metal/metal oxide nanocrystals with thiol modification was used to produce structures with multiscale roughness and controlled wettability, to separate water from chloroform, hexane, hexadecane and edible oil^23^. Similar to sponges, membranes can be used to separate oil from water. Examples of membranes include polymeric filtration membranes and metallic meshes^24^, chitin, cellulose, and carbon nanotubes^25–27^. All of these materials (sponges and membranes) are produced ex-situ, and would not be applicable to produce separation barriers in the subsurface.

While extensive research has been conducted on the development of sponges and filters for separation of oil from surface waters, only one study explored the use of kapok (a plant fiber) to separate NAPL from water in aquifers^28^. Walls produced with this fiber require soil excavation to be installed, leading to technical challenges when used to treat deep contamination^29^. Also, the previous study conducted using kapok did not analyze the effect of oil and emulsifier type on the effectiveness of the kapok wall in separating oil from water. Capillary forces hinder hydrocarbon extraction from the aquifers^30^. Flushing NAPL-contaminated aquifers with emulsifiers is therefore required to mobilize NAPL, promoting their extraction^31,32^. Other previous studies used injectable barriers to contain hydrocarbons, which could be used to ensure that hydrocarbons remain contained while being treated through either chemical or biological remediation methods^29,31,32^. However, these barriers did not allow water flow while containing NAPL. Injectable filters that act as selective permeable barriers (allowing water flow while containing NAPL) could be used in combination with extraction wells and with surfactant flushing. This combination would allow mobilizing NAPL, which would flow towards the semipermeable barrier and be extracted through a pumping well, while water would flow through. Semi-permeable barriers could also be utilized when NAPL are being treated with chemical methods (e.g. Fenton’s reagents^33^ and oxidizers^34–36^) or biological methods (e.g. bioremediation using bacteria^37,38^). NAPL have limited mobility in aquifers when they are not emulsified with surfactants. Nonetheless, it is beneficial to ensure protection of downstream receptors (e.g. potable water wells) during their remediation, e.g. by using selective barriers. Here, we produced injectable filters (selective barriers) with chitosan, hydroxyethylcellulose or cationic hydroxyethylcellulose, to separate organic solvents from water in the subsurface. Specifically, we investigated the effect of solvent polarity and emulsifier type on NAPL-water separation efficiency.

## Experimental section

### Materials

Purified zein, KCl, salt (reagent grade), acetic acid (100%, Glacial, Anhydrous), toluene (HPLC grade, Fischer Chemical), hexane (HPLC grade, Fischer Chemical), Tween 20 (Ultrapure, Thermo Scientific), oleic acid (99.0 +%, TCI America) were purchased from Fisher Scientific. Chitosan (75% deacetylated, ACROS Organics), 2-hydroxyethyl cellulose (HEC) and hydroxyethylcellulose ethoxylate, quaternized (HEC +) were purchased from Sigma Aldrich. Tetrahydrofuran (THF, UN2056) was purchased from Caledon Laboratories Ltd. TCE (ACS, 99.5% min) was purchased from Alfa Aesar. Ethanol (anhydrous, commercial alchols) was purchased from Greefield Global Inc. Nile Red (technical grade, 95% pure) and humic acid sodium salt (abbreviated as humic acids, HA) were purchased from Sigma Aldrich. Ottawa sand was purchased from BEI/PECAL (Stake Technology Ltd.). Deionised (DI) water was used in all experiments. The pH of DI water was approximately pH = 6.

### Zein solution and particle preparation

Purified zein (4/L) was dissolved in DI water at pH = 13 (adjusted with NaOH). The solution was then used as such, or after adding KCl salt to obtain a 12 mM salt concentration. Addition of salts to zein solution produced zein particles (diameter = 113.7 ± 3.3 nm), as described elsewhere^32^.

### Polymer solutions

HEC and HEC + were dissolved in DI water at 0.01 wt% concentrations and allowed to hydrate for 1 h, while periodically hand-mixing with a spatula. Chitosan was dissolved in 5% (volume based) glacial acetic acid (GAA) solution at pH = 4, also at 0.01 wt% concentrations, and hydrated for 5 min (chitosan hydrated faster in GAA solution at pH = 4 compared to HEC and HEC + in DI water at pH = 6).

### Filter preparation

Ottawa sand was inserted dry (without packing it) in a 10 mL graduated glass cylinder having a diameter of approximately 1 cm and perforated at the bottom with a 0.5 cm hole. The volume of Ottawa sand used was 2 mL (including void spaces). Sand was flushed with DI water, before injecting 1 mL of 0.01 wt% polymer solution (HEC, HEC + or chitosan) on top of the sand bed. After polymer injection, the sand column was profusely rinsed with DI water (using 10 volumes or more of the column volume, with the aid of plastic pipette) to remove excess polymer. The filters were then immediately used for filtration experiments.

Filters were also obtained with dry chitosan inserted into a 10 mL glass cylinder perforated at the bottom and lined with filter paper (to prevent chitosan from passing through the perforated bottom of the cylinder). The volume of dry chitosan used was 2 mL (including void spaces). These filters barriers were not wetted with water before use.

### Filtration experiments

Four types of filtration experiments were conducted. In the first type of experiments toluene, hexane, THF or TCE (4 mL, without Nile Red) were pipetted using a glass pipette on top of the injectable filter (which was pre-washed with water, but from which water was drained), followed by addition of DI water (6 mL) on top of the filter. In experiments conducted with hexane and toluene, water eluted from the filter was collected in a glass container and immediately transferred into gas tight vials for analyses using gas chromatography–mass spectroscopy (“Gas chromatography–mass spectroscopy (GC–MS)” section). Water eluted from the filter was not analyzed in experiments conducted with THF and TCE, because THF and TCE flowed through the column (as determined with the naked eye). The second set of experiments conducted was identical to the first one, except that toluene and hexane were dyed with Nile red (2.5 × 10^–3^ and 5 × 10^–3^ g/L of solvent, respectively). The water eluted from columns was immediately analyzed using fluorescence spectroscopy (“Steady state fluorescence spectroscopy measurements” section). In the third type of experiment toluene or hexane (4 mL) were emulsified with DI water (6 mL). The emulsifiers used were Tween 20 (2.5 wt%), oleic acid (100 μL in 6 mL DI) and humic acid (HA, 1 g/L), added to DI water at pH = 6. Zein was also used as emulsifier at pH = 13, with or without KCl (as described in “Zein solution and particle preparation” section). The volume of hexane and toluene eluted from the filter was quantitated with a glass graduated cylinder. In the fourth type of experiments either DI water (6 mL) or hexane, toluene, THF and TCE (6 mL) were pipetted on top of a dry chitosan filter.

### Gas chromatography–mass spectroscopy (GC–MS)

The concentrations of toluene in the water eluted from the filters were determined using a 6890N Network Gas-Chromatography system (Agilent Technologies) equipped with a 6783B series injector. Samples of water eluted from the column were diluted with 50% ethanol before each analysis, and analyzed using liquid injection. The R^2^ values of the calibration curves were 0.99 or greater. The toluene peaks were quantified using Enhanced Data Analysis software (Agilent Technologies).

### Steady state fluorescence spectroscopy measurements

Steady state fluorescence spectroscopy was used to determine the concentrations of toluene and hexane in water eluted from the filters using a FluoroMax-4 spectrofluorometer (Horiba Scientific Inc., Edison, NJ, USA). Water samples were diluted with ethanol (50% ethanol, volume based) and loaded in 1 cm light path quartz cuvettes (Firefly Sci, Staten Island, NY, USA). Due to hexane’s lack of autofluorescence and to facilitate detection of toluene, Nile red was added to these solvents. Samples were excited at 525 nm, and the spectra were collected over an emission range from 550 to 800 nm. The excitation and emission slits were set to 3 and 10, respectively. The Nile Red fluorescence intensity was correlated to the hexane and toluene concentrations using the calibration curves shown as supplementary information (Figs. SI.1 and SI.2). The toluene and hexane solutions containing Nile red (2.5 × 10^–3^ and 5 × 10^–3^ g/L of solvent, respectively) were diluted with ethanol to different concentrations to obtain the calibration curve (R^2^ > 0.96, Figs. SI.1 and SI.2, supplementary information). Control samples were also tested to eliminate background contributions.

### Quartz-crystal microbalance with dissipation monitoring (QCM-D)

A QCM-D system (Q-Sense, Biolin Scientific, Sweden) was used to investigate the sorption of chitosan, HEC and HEC + onto silica. In QCM-D experiments, the solution of interest (HEC, HEC + and chitosan solutions) flowed through a flow cell (approximately 5 mm thick) under laminar flow conditions. The concentrations used were 0.1 wt% of either HEC or HEC + in DI water at pH = 6, or 0.01 wt% chitosan in 5% GAA solution at pH = 4. Experiments were conducted at a flow rate of 10 ml/min and at a temperature of 23 °C. The QCM-D sensor (located at the bottom of the flow cell) was an AT-cut quartz crystal, sandwiched between two electrodes. The AT-cut quartz crystal is a piezoelectric material (i.e. it deforms when a differential voltage is applied across its two faces). Therefore, when an alternating differential voltage was applied between the electrodes, the AT-cut sensor oscillated. The top of the electrode was sputter-coated with silica, which formed a coating that was strongly affixed onto the sensor surface and moved with it. Silica coated sensors were purchased from Q-Sense (product id. QSX 303). At the start of each measurement, the resonant frequency F and the overtones (odd multiples of the resonant frequency) were measured in the background solution (DI water) to obtain a stable baseline. Following stabilization, the polymer solutions were injected in the flow cell (Table 1). Upon injecting the solution of interest (i.e. polymer solutions), shifts of the resonant frequency and the overtones were recorded. Changes of the resonant frequency and the overtones can be related to the deposition of films onto the sensors^39^. The dissipation factors D (which related to film softness^40^) were also determined.

**Table 1:** Injection sequence during QCM-D experiments.

Step	Injection time (min)	Solution
1	10	Deionised water
2	5 (chitosan)	Polymer solution (chitosan, HEC or HEC +)
	10 (HEC and HEC +)	
3	10	Deionised water

It is noted that in QCM-D experiments silica-coated sensors were selected to mimic Ottawa sand (used in filtration experiments). Ottawa sand could not be placed loosely on the sensor, because the sensor coating must move in perfect synchrony with the sensor, to ensure that changes of the resonant frequency and the overtones are correlated exclusively to polymer deposition onto the sensor surface. Also, coatings on the QCM-D sensors must be thin, to avoid losing sensitivity. Since the motion of the sensors is very small (30 nm/V)^41^, it does not disturb deposition or film structure.

### Contact angle measurements

Contact angles were measured using a Biolin Scientific Theta Lite optical tensiometer (TL, Finland) with Attension software (Biolin Scientific, v 2.1). Static contact angles were measured in air using DI water, toluene and hexane. The substrates used were glass slides coated with chitosan (12 g/L in 5% GAA), HEC and HEC + (12 g/L in DI water at pH = 6), air dried before each measurement. Sliding contact angles were measured in water using glass cuvettes. The bottoms of the glass cuvettes were coated with chitosan, HEC or HEC + by pipetting on it the polymer solutions (0.01 wt% chitosan solution in 5% GAA, or 0.1 wt% of either HEC or HEC + in DI) and subsequently rinsing it with DI after 1 min contact time. The cuvettes were then filled with DI and a droplet of the solvent (toluene, hexane or TCE) was contacted onto the polymer coated bottom of the cuvette with the aid of a glass Pasteur pipette.

### Shear rheology experiments (strain sweeps)

The shear rheology of HEC and HEC + was studied at 23 °C in water (pH = 6) and in water–ethanol mixtures (with 0.5%, 1% and 5% ethanol, volume based) after hand-mixing for 120 s. Strain sweeps were conducted using a rotational torque-controlled (i.e. combined-motor-transducer type) rheometer (MCR302 Anton Paar, Graz, Austria), with a single-gap concentric-cylinder geometry (inner radius = 13. 33 mm and height = 40 mm). During strain sweeps, the frequency was constant (6 rad/s) and the strain was increased from 0.01 to 1,000%. The values of the elastic modulus G′ and the viscous modulus G″ are reported for the linear viscoelastic region, where they were independent of strain.

## Results and discussion

Polymer-based injectable filters relied on polymer sorption onto the geological substrates in aquifers (soil or rock), to produce a porous medium able to exclude organic solvents, while allowing water flow. Chitosan, HEC and HEC + were pre-selected as preferred polymers because we hypothesized that they would be oleophobic upon immersion in water. QCM-D experiments demonstrated their sorption onto silica, chosen as model geological substrate (“Effect of solvent polarity on filtration” section). Contact angles measured between polymer coated surfaces immersed in water and organic solvents demonstrated the oleophobicity of surfaces coated with these polymers (“Effect of solvent polarity on filtration” section). Separation efficiency between organic solvents and water depended on the polarity of the organic solvents (“Effect of solvent polarity on filtration” section) and on the emulsifiers added (“Effect of emulsifier on filtration” section). These results are discussed in the following sections.

### Polymer sorption onto mineral surfaces

Sorption of chitosan, HEC and HEC + onto silica was verified with QCM-D experiments (Table 2). QCM-D experiments showed that the third overtones (and all others) decreased upon injection of polymer solution, indicating sorption^39,42^. Upon rinsing the cell with DI water following polymer injection, the overtones did not return to the baseline values, indicating that the polymers remained adsorbed at the silica surface^39,42^. The dissipation factor increased upon polymer injection, but the ratio between the dissipation factor and the overtones was D/F < 1. The dissipation factor is related to how rapidly the wave propagating through the crystal sensor dissipates, when the sensor is no longer oscillated (i.e. when no alternating differential voltage is applied across it). High D/F ratios are indicative of soft films, which rapidly dissipate the propagating wave. Ratios of D/F < 1 are indicative of fairly rigid films^39^. Chitosan was previously adsorbed onto sand activated with HCl and high temperature (90 °C)^43^, onto untreated sand at pH = 4, followed by pH neutralization with NaOH^44^, or onto porous silica^45^. The pKa of chitosan is ~ 6.20 and thus at pH = 4 it is 99% protonated^46^. The point of zero charge of silica (sand) is at pH = 2–3^47^, and silica should be therefore be negatively charged at pH = 4. Electrostatic attraction should have therefore contributed to sorption of chitosan onto sand. After flushing with DI water (pH ≈ 6), the degree of protonation should have decreased by 50%, inducing partial desorption of chitosan but nonetheless allowing electrostatic attraction between the portion of protonated chitosan sites and silica. These considerations explain in part the increase of ΔF from − 28 to − 4.2 Hz. In addition to desorption, bulk effects should have also contributed to this increase^39^.

**Table 2:** Values of the third overtone F and its related dissipation factor (D) relative to the baseline measured in DI water during QCM-D experiments.

	chitosan		HEC		HEC +	
	ΔD (1 × 10^–6^)	ΔF (Hz)	ΔD (1 × 10^–6^)	ΔF (Hz)	ΔD (1 × 10^–6^)	ΔF (Hz)
DI baseline	0.0	0.0	0.0	0.0	0.0	0.0
Polymer + DI	11.2	− 28.0	3.5	− 9.4	9.7	− 41.7
DI rinse	0.8	− 4.2	1.3	− 8.7	8.0	− 40.2

Other overtones and dissipation factors have different values but followed similar trends (they are not reported here for brevity).

QCM-D data indicate that HEC and HEC + could also adsorb onto silica and did not desorb upon rinsing with DI water, as shown by the shift in the overtones (Table 2). These shifts were more significant with HEC + than with HEC, suggesting greater HEC + sorption. This result is attributed to the increased electrostatic attraction between the polymer and silica, because of the positive charge of the N(CH_3_)_3_ groups of HEC +.

However, electrostatic attraction alone cannot facilitate the sorption of HEC + onto silica. HEC and HEC + have a similar structure, with the exception of the cationic group on HEC +, and HEC could also adsorb onto silica. HEC sorption onto silica has been previously reported and attributed to hydrogen bonding^48,49^, which also likely contributed to HEC + sorption onto silica.

In addition to sorption onto surfaces, QCM-D also provides information regarding film structure^39^. When the absolute value of the ratio DD/DF < 1 × 10^–6^ Hz^−1^, films are compact and fairly stiff^50^. After rinsing with the DI, the absolute values of DD/DF ratios were less than 2 × 10^–7^ Hz^−1^ for all systems, indicating compact films.

### Effect of solvent polarity on filtration

**Figure 1:**
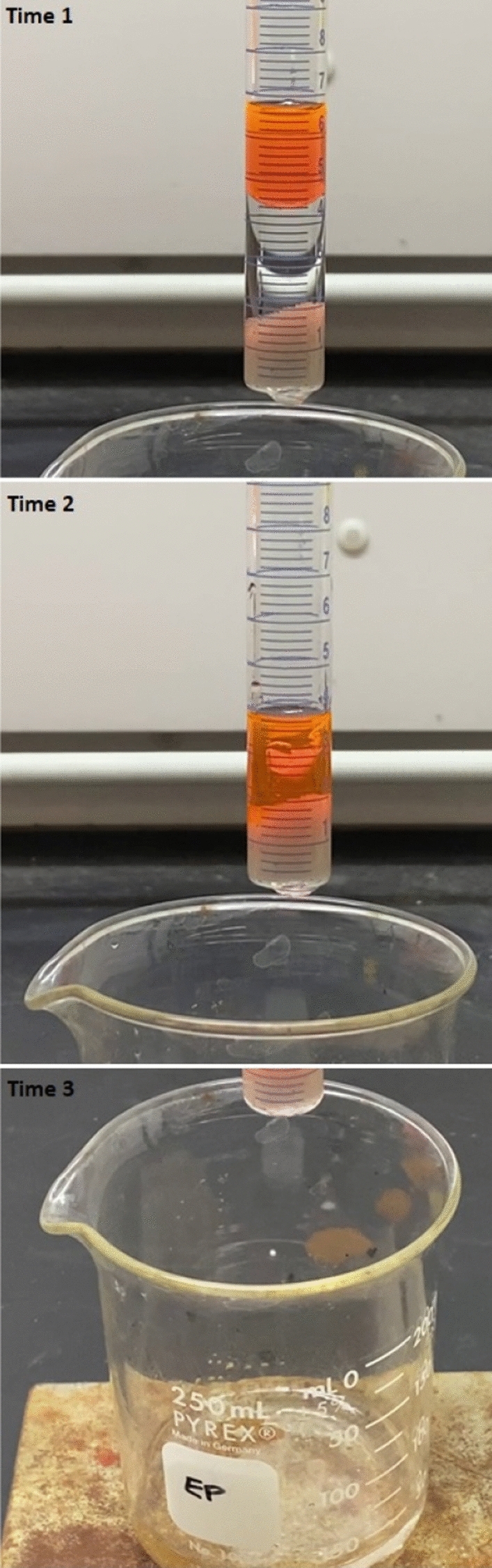
A chitosan filter in a glass cylinder perforated at its bottom. The images show the temporal sequence of water flow, followed by toluene retention after all water eluted from the filter. The glass beaker where eluents were collected contained water only. Toluene was dyed with Nile red (for visual contrast). Similar results were obtained with chitosan and hexane, and with HEC and HEC +, with either toluene or hexane. This image was obtained by Erica Pensini.

In the absence of emulsifiers, toluene and hexane were effectively retained by the chitosan, HEC and HEC + filters (selective barriers) obtained by injecting aqueous solutions of these polymers into beds of sands. Instead, water flowed through these filters (Fig. 1 and Video 1). When water (6 mL) and toluene (4 mL) were pipetted on top of these filters, the concentrations of toluene and hexane in the water eluted from the filter were at the ppm level (Table 3 and Fig. SI.3). The concentrations of toluene in water determined with fluorescence spectroscopy were higher than those determined with GC–MS, possibly because the solvent contained Nile Red. TCE and THF flowed through these filters, similar to water.

**Table 3:** Toluene and hexane concentrations in water eluted from chitosan, HEC and HEC + filters obtained using sand, as determined with GC–MS and fluorescence spectroscopy.

	Toluene, GC–MS (ppm)	Hexane with Nile Red, fluorescence spectroscopy (ppm)	Toluene with Nile Red, fluorescence spectroscopy (ppm)
Chitosan	4.05	98	39
HEC	4.27	114	17
HEC +	3.17	165	25

Oil–water separation sponges with good performance were previously synthesized. For instance, polyurethane foams reinforced with carbon nanotubes could adsorption up to 34.5 g/g of oil, retaining high adsorption performance after being reused 150 times^51^. Polyurethane sponges modified with (3-Mercaptopropyl)trimethoxysilane and graphene oxide achieved an oil separation efficiency greater than 99.5%^52^. Methyltrichlorosilane salinized sponges had an adsorption capability of diesel of 65 g/g^53^. Polymethylsilsesquioxane modified sponges could remove up to 58–127 g/g of diverse oils (e.g., diesel, pump oil, hydraulic oil, and transformer oil) from water^54^. Melamine-based sponges coated with hydrophobic lignin shells had a sorption capacity of 18–51 g/g for various oils^55^. Superhydrophobic kaolinite modified graphene oxide-melamine sponge could remove dimethylformamide (89 g/g) and diesel oil (76 g/g)^56^. Sponges have also been used to separate surfactant-stabilized emulsions. Superhydrophobic attapulgite coated polyurethane sponges could separate Tween 80-stabilized oil-in-water emulsions obtained using kerosene, diesel, petroleum, toluene, and *n*-hexane, with an efficiency up to 99.87%^57^. Polyurethane-graphene sheet sponges could separate emulsions obtained with hexane, hexadecane, and soybean with an efficiency greater than 90%^58^. Additional examples of sponges can be found in a review on the subject^13^. Sponges previously develop and described above sorbed (rather than filtered) oil. Effective oil–water separation membranes have also been developed. For instance, oil–water separation membranes produced using 2-(Dimethylamino)ethyl methacrylate (DMAEMA) and 4-vinylbenzyl chloride (VBC) had a separation efficiency greater than 90%^59^. Fluorinated carbon nanotubes were deposited onto carbon fabrics to create hierarchical oil–water separation membranes with 99.7% separation efficiency^60^. Titanium oxide oil–water separation membranes achieved a separation of 99.2%^61^. Wood-free fiber-based 2,2,6,6-tetramethylpiperidine-1-oxyl oxidized cellulose nanofiber were used to increase the hydrophilicity and underwater oleophobicity of cellulose sponges, to achieve an oil–water separation efficiency of over 90%^62^. While effective, both sponges and oil–water separation filters previously developed cannot be injected in porous media (such as aquifers) to separate oil from water in the subsurface. Instead, our filters offer the advantage of being injectable around polluted areas, and are therefore well-suited to confine solvents such as hexane and toluene during groundwater remediation. Future research should focus on developing injectable filters to also separate other solvents such as TCE, which could not be separated from water using our filters.

Differences in the retention of the solvents are attributed to solvent polarity, which increased in the following order (Table 4): water > THF > TCE > toluene > hexane. While organic, non-polar solvents such as toluene and hexane were retained, organic but polar solvents such as TCE and THF could flow through the filters, similar to water.

**Table 4:** Polarity of the solvents used in this study^63^.

Solvent	Polarity E_T_ (kcal/mol)
*n*-hexane	30.9
Toluene	33.9
Tricholoroethylene, TCE	35.9
Tetrahydrofuran, THF	37.5
Water	63.1

**Figure 2:**
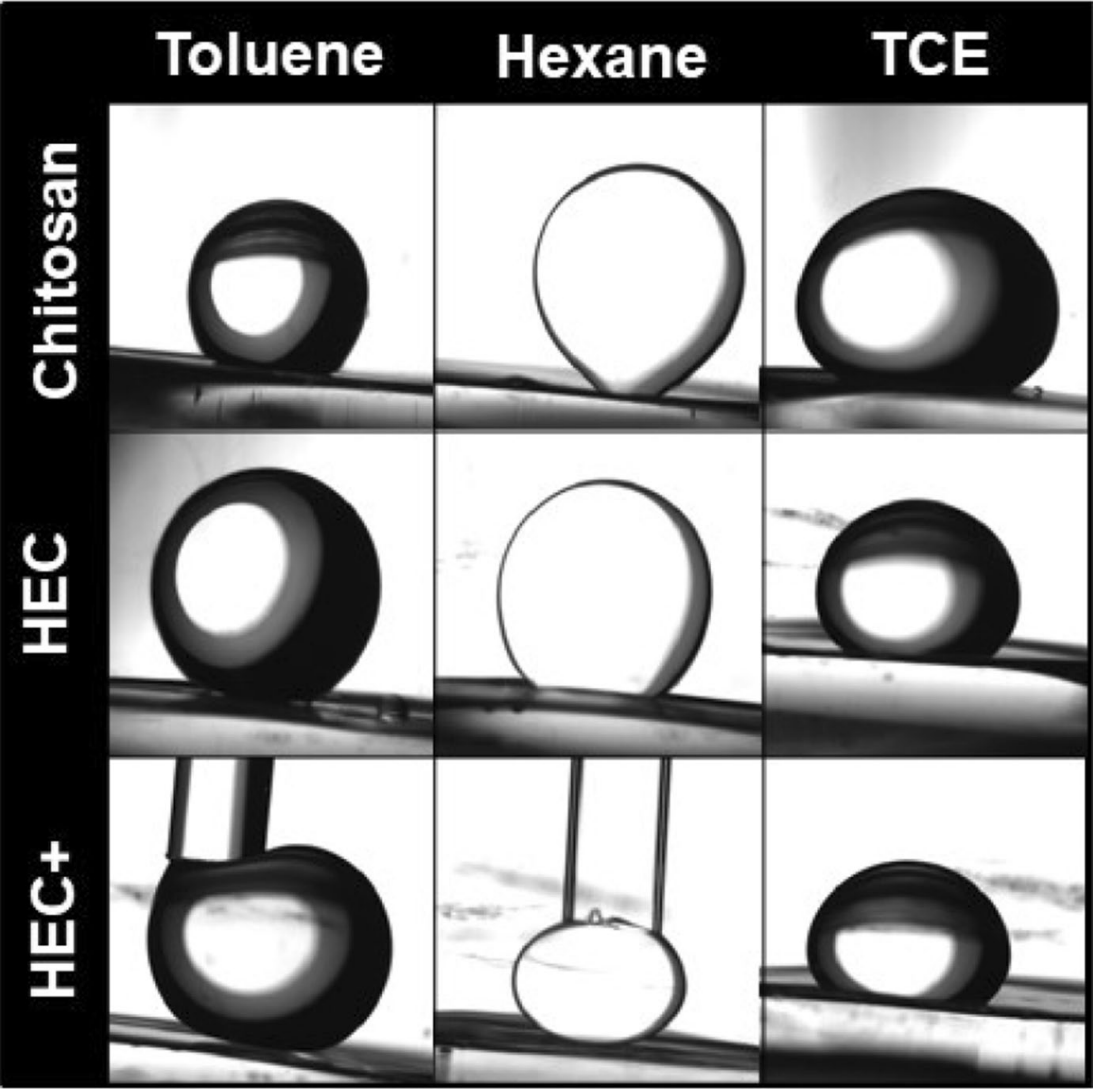
Sliding contact angle of hexane, toluene and TCE with chitosan, HEC and HEC + films in DI water. Images were obtained by Erica Pensini and Tatianna Marshall using a Biolin Scientific Theta Lite optical tensiometer (TL, Finland) with Attension software (Biolin Scientific, v 2.1, https://www.biolinscientific.com/attension/optical-tensiometers/theta-lite).

Contact angles were measured to gain insights regarding the affinity of chitosan, HEC and HEC + coated surfaces for the organic solvents used. In air, the static contact angle formed by a water droplet was 40.1° ± 6.9° for chitosan, 33.8° ± 4.7° for HEC and 20.3° ± 2.1° for HEC +. Instead, hexane and toluene fully wetted dried films of chitosan, HEC and HEC + (i.e. the contact angles were zero). In water, chitosan, HEC and HEC + films were oleophobic, with sliding contact angles were > 90° for toluene, hexane and TCE, as shown in Fig. 2 (measurements were not conducted with THF, which is miscible in water). A previous study showed that shrimp shells (containing chitin) and cross-linked chitosan films had low contact angles with either water or crude oil in air, but had super-oleophobic behavior in water, where crude oil formed high contact angles with them^64^. This study also showed that filters obtained by crosslinking chitosan with glutaraldehyde on a metal mesh could retain crude oil, while allowing water flow^64^.

**Figure 3:**
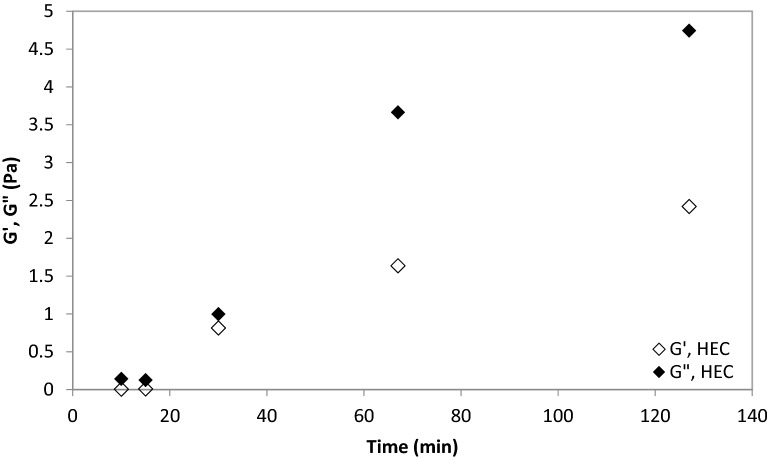
Viscous (G″) and elastic (G′) moduli of HEC (12 g/L) solutions in water over time.

**Figure 4:**
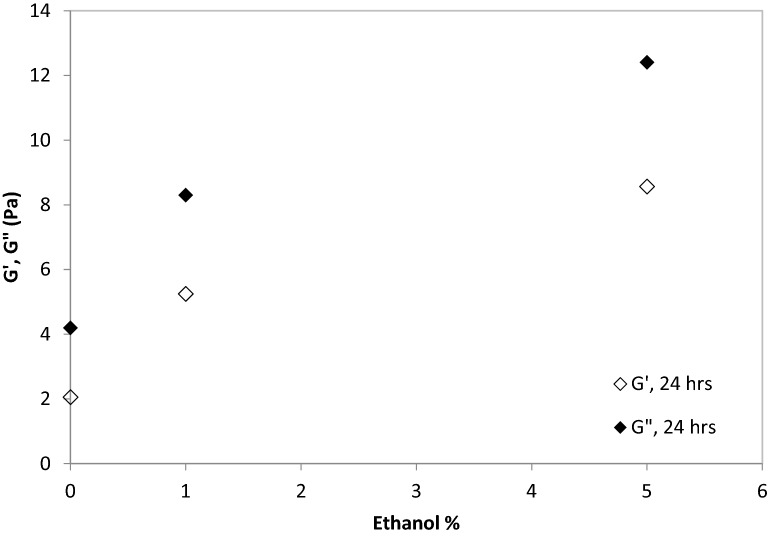
Viscous (G″) and elastic (G′) moduli of HEC (16 g/L) solutions in water containing different percentages of ethanol (volume based), measured after 24 h.

Super-oleophobic surfaces can be sticky or non-sticky^65–67^. In our study, toluene and hexane formed contact angles > 90° with chitosan, HEC or HEC + films, but they could stick (adsorb) exclusively on chitosan and HEC films. Instead, HEC + films repelled hexane and toluene, and droplets had to be kept in contact in them with the aid of a glass pipette, to prevent detachment (Fig. 2). Chitosan is insoluble in water at neutral pH, whereas HEC and HEC + can completely dissolve in water. These results suggest that chitosan is the least hydrophilic of the polymers used. Its low affinity for water can explain why toluene and hexane could stick onto chitosan-coated surfaces, even though their contact angles with chitosan coated surfaces in water were > 90°. Hydrophobicity and oleophobicity are not contradictory attributes, and surfaces able to repel oils, usually also usually repel water^65^. For instance, polyester fabric and polyurethane sponges treated with heptadecafluorononanoic acid could repel both water ions and oil^68^. HEC behaved similarly to chitosan (hexane and toluene could stick onto its surface, but their contact angles were > 90°). While HEC could dissolve in water, the hydration time was significant, as reflected in the slow increase in the shear viscoelastic moduli over time (Fig. 2). In good solvents polymers acquire a stretched conformation^39^, which leads to entanglement between polymers and to an increase in their viscoelastic moduli^69,70^. Addition of ethanol (up to 5%, v/v) increased the magnitude of the viscoelastic moduli relative to pure water (Figs. 3, 4). This result is attributed to the increased hydrogen bonding with low ethanol concentrations, due to the formation of transient ethanol hydrates^71^. This result also suggests incomplete HEC hydration in pure water (and more complete hydration in ethanol–water mixtures). Hydrogen bonding was disrupted in ethanol without water, and HEC did not dissolve. A non-monotonic viscosity change upon increasing the ethanol concentration in water was observed for other cellulose ethers, and attributed to increased hydrogen bonding up to a critical ethanol concentration in water, above which hydrogen bonding was disrupted^72^. This result suggests that although HEC is oleophobic (the contact angles with hexane and toluene are > 90°), its hydration in pure water was not complete, possibly explaining oil sticking. Toluene and hexane formed contact angles > 90 with HEC +, and they could not stick onto it. This result suggests that HEC + films were more oleophobic than HEC and chitosan films. The increased oleophobicity of HEC + modified with a quaternary ammonium group is in agreement with previous studies, which report that this modification increased the hydrophilicity (and hence the oleophobicity) of HEC^73^. After 3.5 h the shear viscoelastic moduli of 20 g/L HEC + were similar without ethanol and with ethanol concentrations up to 5% (G′ below detection; G″ $\approx$ 1.5 Pa), whereas HEC + did not dissolve in pure ethanol. These results suggest that hydration of HEC + was complete in pure water, dissimilar to HEC. Also, the viscoelastic moduli of 20 g/L HEC + were similar between 30 and 127 min (G′ below detection; G″$\approx$1 Pa), suggesting that hydration was complete after 30 min (and hence faster compared to the hydration of HEC).

As mentioned earlier, TCE was not retained by filters obtained by coating sand with either chitosan, HEC or HEC +. TCE has a density of 1.46 g/mL at 20 °C, which is higher than the density of water. However, we contend that TCE flow through the filters is not due to the high TCE density. THF could also flow through the filters, but its density (0.889 g/mL at 25 °C) is lower than the density of water. Similar to hexane and toluene, TCE formed contact angles > 90° with chitosan, HEC and HEC +. The contact area between TCE and either chitosan, HEC and HEC + was greater than the contact area between either hexane and toluene and the polymer. The high density of TCE and the weight of TCE droplets can certainly affect the contact area. Nonetheless, the contact area between TCE and HEC + was greater than that of hexane and toluene droplets pressed against HEC + with a glass pipette. This result suggests that TCE has higher affinity for HEC + than for toluene or hexane, likely due to its polarity. The contact area between toluene (more polar than hexane) and HEC + was greater than the contact area between hexane and HEC +, even when droplets were compressed against its surface (overriding density differences and buoyancy effects). We speculate that TCE also has higher affinity for HEC and chitosan compared to hexane and toluene, likely explaining why it could flow through polymer coated sand filters, similar to THF and water. Additional research should be conducted to further understand the interactions between TCE and THF, and chitosan, HEC and HEC +.

When hexane or toluene were pipetted on dry chitosan, their flow was not impeded. Instead, water pipetted on dry chitosan could not flow. This result is attributed to the oleophilicity of dry chitosan and is in agreement with previous studies, which show that shrimp shells (mainly constituted of chitin, from which chitosan is obtained) are oleophilic in air, and superoleophobic in water^64^.

In summary, since our filters (selective barriers) do not allow the flow of non-polar NAPL, they would provide protection for downstream receptors during NAPL remediation with biological or chemical remediation methods. The next section discusses the integration of selective barriers with physical remediation methods, such as surfactant flushing.

### Effect of emulsifier on filtration

The type of emulsifier used strongly affected the effectiveness of chitosan, HEC and HEC + coated sand filters (selective barriers) in retaining toluene and hexane emulsified in water, as summarized in Table 5.

**Table 5:** Retention of toluene and hexane emulsified in water using different emulsifiers.

Emulsifier	pH	Separation
Tween 20 (pH = 6)	6	No separation
Oleic acid	6	No separation
Zein solution	13	No separation
Zein particles (obtained with KCl)	13	62% (volume based) retention of toluene and hexane
Humic acids	5.5	> 97% (volume based) retention of toluene and hexane

The results were similar for all filters.

Classical surfactants with a hydrophobic tail and a hydrophilic head prevented oil–water separation, regardless of their HLB (hydrophilic lipophilic balance) number. The HLB number is related to the hydrophilicity of surfactants, with high HLB numbers indicating high hydrophilicity^31^. Tween 20 (HLB = 16.7^74^) is hydrophilic and oleic acid (HLB = 1^75^) is hydrophobic. Regardless of their HLB number, classical surfactants should orient themselves with the hydrophilic head exposed to water and the hydrophobic tail exposed to oil (toluene or hexane). Therefore, hexane and toluene droplets in water should be surrounded by a layer that appears hydrophilic to an observer located in water. This hydrophilic layer should control the interactions between the oil droplets and surfaces immersed in water, e.g. polymer coated sand filter surfaces. Flow of hexane and toluene droplets through the filter with Tween 20 and oleic acid is attributed to the hydrophilicity of the surfactant head layer. If filters were flushed first with Tween 20 and then with pure water, they separated toluene and hexane from water (without additional emulsifiers added). This observation indicates that either surfactants did not adsorb onto the filters or that sorption was reversible.

Zein and humic acids were also tested for two reasons. The first reason is that they are an example of non-classical surfactants. Classical surfactants have a hydrophilic head and a hydrophobic tail, whereas humic acid and zein have more complex structures. Also, the hydrophilicity of zein in water can be tuned by adding salts (e.g. KCl), as previously described and will be further discussed below^32^. Using zein and humic acids therefore allows to assess the effect of the surfactant structure (classical, with a head and a tail, vs. non-classical) and the hydrophilicity of the hydrocarbon-water interface on the effectiveness of selective filters in separating water from emulsified solvents. The second reason for which humic acid and zein were used is that they can have useful applications in groundwater remediation. As previously mentioned, one strategy to remediate hydrocarbon spills in polluted aquifers is to extract them using pumping wells^32^. Surfactants may be used to emulsify hydrocarbons (NAPL), facilitating their extraction from polluted aquifers. Natural emulsifiers (such as zein and humic acids) offer advantages over synthetic surfactants, which can harm bacteria in the aquifers^30^. Since bacteria have the ability to degrade hydrocarbon residuals in groundwater, using natural emulsifiers is preferable^30^. Our previous studies showed that zein^32^ and humic acids^30^ can emulsify hydrocarbons in water, and are therefore promising alternatives to synthetic surfactants. Therefore, zein and humic acids were used here to verify if they could be utilized in combination with our selective filters, to facilitate hydrocarbon extraction while ensuring their containment within the treated area (during groundwater remediation).

Zein is soluble in water at alkaline pH, and it can solubilize hydrocarbons when it is solubilized^32^. Salts such as KCl ‘salt out’ zein, forming water-insoluble nano-sized aggregates (particles), which can stabilize emulsions through Pickering stabilization mechanisms at pH = 13^32^. Zein aggregation with salts is attributed to its increased hydrophobicity^32^. The oil–water interface of hexane and toluene droplets surrounded by zein dissolved in water (at pH = 13, without KCl) is more hydrophilic than the surface of oil droplets surrounded by zein particles (at pH = 13, with KCl). As discussed for Tween 20 and oleic acid, flow of hexane and toluene droplets stabilized by dissolved zein is ascribed to the hydrophilicity of the oil–water interface. Zein particles adsorbed at the oil–water interface likely improve oil–water separation by increasing the hydrophobicity of oil–water interfaces. Zein particles could flow through the filters without slowing the flow, and after flushing with pure water the filters could separate oil from water (in the absence of added emulsifiers). These results strongly suggest that partial oil–water separation with zein particles was not due to clogging of the filters or to irreversible zein particle sorption onto polymer coated sand filters.

Humic acids stabilize diluted bitumen in water because they comprise both hydrophobic and hydrophilic moieties^30^. Dissimilar to classical surfactants, humic acids have a complex structure rather than a single hydrophilic head and a single hydrophobic tail, as indicated above. Therefore, humic acids cannot likely form oil–water interfacial films that are as hydrophilic as films formed by classical surfactants (e.g. Tween 20 and oleic acids), relative to an observer (or another body, e.g. the filter surface) located on the water side. Partial oil–water separation with humic acids is ascribed to the partial hydrophilicity of the side of humic acid interfacial films exposed to water, which interacts with the polymer-coated sand filter surface.

In summary, the data strongly suggest that oil–water separation is correlated with the characteristics of the stabilizing interfacial films adsorbed at the interface between water and hexane and toluene. No separation occurs if the surface of interfacial films exposed to the water side is hydrophilic. Separation is improved by increasing the hydrophobicity of the side of interfacial films exposed to water, because this side of the films interacts with the surface of the filters. While the data suggest that in this study the hydrophobicity of the droplets control the effectiveness of emulsion separation, charge can also play a role. A previous study conducted with positively and negatively charged hydrophilic membranes investigated separation of oil in water emulsions stabilized by sodium dodecyl sulfate (SDS, anionic surfactant) and cetyltrimethylammonium bromide (CTAB, cationic surfactant)^76^. Positively charged membranes were effective at intercepting SDS, leading to low TOC (total organic carbon, used to quantitate the amount of oil in water), while the TOC was higher when CTAB was used as surfactant^76^.

Our results show the importance of selecting the correct emulsifier, if the selective barriers described in this paper are used in the subsurface to aid the remediation of NAPL spills, in conjunction with surfactant flushing. It is envisioned that solvents dispersed in groundwater with suitable emulsifiers would migrate toward the selective barriers, where they would collect and be easily pumped.

## Conclusions

We developed NAPL-water separation filters that can be used to obtain subsurface selective barriers in aquifers (allowing exclusively water flow, while stopping non-polar NAPL). Our filters are composed of sand coated with chitosan, HEC and HEC +. Aqueous solutions of these polymers adsorb onto wet sand, and can be injected in sandy aquifers to form oil–water separation barriers. Selective barriers were effective in separating water from non-polar organic solvents, such as hexane and toluene. They can therefore be utilized in conjunction with chemical or biological remediation methods, to ensure NAPL containment during their remediation, for the protection of downstream receptors (e.g. potable water wells). Instead, selective barriers could not separate water from polar organic solvents, such as THF and TCE, for which alternative filtration systems should be developed in future studies. When toluene and hexane were emulsified with water, separation efficiency depended on the emulsifier used. Classical surfactants (e.g. Tween 20 and Oleic acid) and zein dissolved in water (without KCl) prevented separation. This is likely because they formed stabilizing interfacial films which were completely hydrophilic on the side exposed to water, hence behaving like water when interacting with the water-wetted surfaces of filters. Over 97% of hexane and toluene were separated from water with humic acid and over 60% with zein particles (formed with KCl addition). These emulsifiers formed stabilizing interfacial films, which were only partially hydrophilic on the side exposed to water. Our future research will focus on developing and identifying emulsifiers that can be used for surfactant flushing (used for the remediation of subsurface hydrocarbon spills) and also allow complete NAPL-water separation with our selective barriers. The use of our selective barriers in non-sandy soils will also be explored.

## Supplementary information

Supplementary Legend.

Supplementary Video 1.

Supplementary Information.

## Data Availability

Additional datasets generated during and/or analysed during the current study are available from the corresponding author on reasonable request.
