# Supplementary material for: Selective solvent filters for non-aqueous phase liquid separation from water
Source: Sci Rep. 2020 Jul 20;10:11931. doi: 10.1038/s41598-020-68920-4 (PMC7371871; doi:10.1038/s41598-020-68920-4)
Supplement: Supplementary file 1 — Supplementary Legend. [file 41598_2020_68920_MOESM1_ESM.docx]

**Video 1** Toluene-water separation with a chitosan filter in a glass cylinder perforated at the bottom. Similar results were obtained with chitosan and hexane, and with HEC and HEC+, with either toluene or hexane. The video to which this still refers is available as supplementary material, video SI.1. This video was obtained by Erica Pensini.
