# Supplementary material for: Selective solvent filters for non-aqueous phase liquid separation from water
Source: Sci Rep. 2020 Jul 20;10:11931. doi: 10.1038/s41598-020-68920-4 (PMC7371871; doi:10.1038/s41598-020-68920-4)
Supplement: Supplementary file 3 — Supplementary Information. [file 41598_2020_68920_MOESM3_ESM.docx]

**Supporting Information for the Manuscript “****Selective Solvent Filters for Non-Aqueous Phase Liquid Separation from Water”**

Tatianna Marshall^1^, Klaudine M. Estepa^1^, Maria Corradini^2,3^, Alejandro G. Marangoni^2^, Brent Sleep^4^, Erica Pensini^1*^

^1^University of Guelph, School of Engineering, 50 Stone Road East, Guelph (ON), N1G 2W1, Canada

^2^University of Guelph, Food Science Department, 50 Stone Road East, Guelph (ON), N1G 2W1, Canada

^3^University of Guelph, Food Science Department, Ontario Agricultural College, 50 Stone Road East, Guelph, ON N1G 2W1, Canada

^4^ University of Toronto, Civil & Mineral Engineering Department, 35 St George St, Toronto (ON), M5S 1A4, Canada

*Corresponding author: email: epensini@uoguelph.ca; phone: +1 519-824-4120 ext. 56746

This supporting information file contains the calibration curve used to quantify toluene and hexane concentrations in the water eluted from filters prepared by injecting chitosan, HEC or HEC+ on a sand bed, followed by rinsing with DI water (Figs. SI.1 and SI.2). The fluorescence spectra are also included (Fig. SI.3).

A sample supporting video demonstrating the separation of toluene from water using a chitosan filter is also available online (although it is not embedded in this file).

**Figure SI.1** Calibration curve used to quantitate hexane concentrations in water eluted from the filters.

**Figure SI.2** Calibration curve used to quantitate toluene concentrations in water eluted from the filters.







**Figure SI.3** Fluorescence spectra of eluted Nile Red in water samples from chitosan, HEC and HEC+ filters and either toluene (left) or hexane (right) as oils. Toluene and hexane contained Nile red, which was used to quantitate their respective concentrations.
